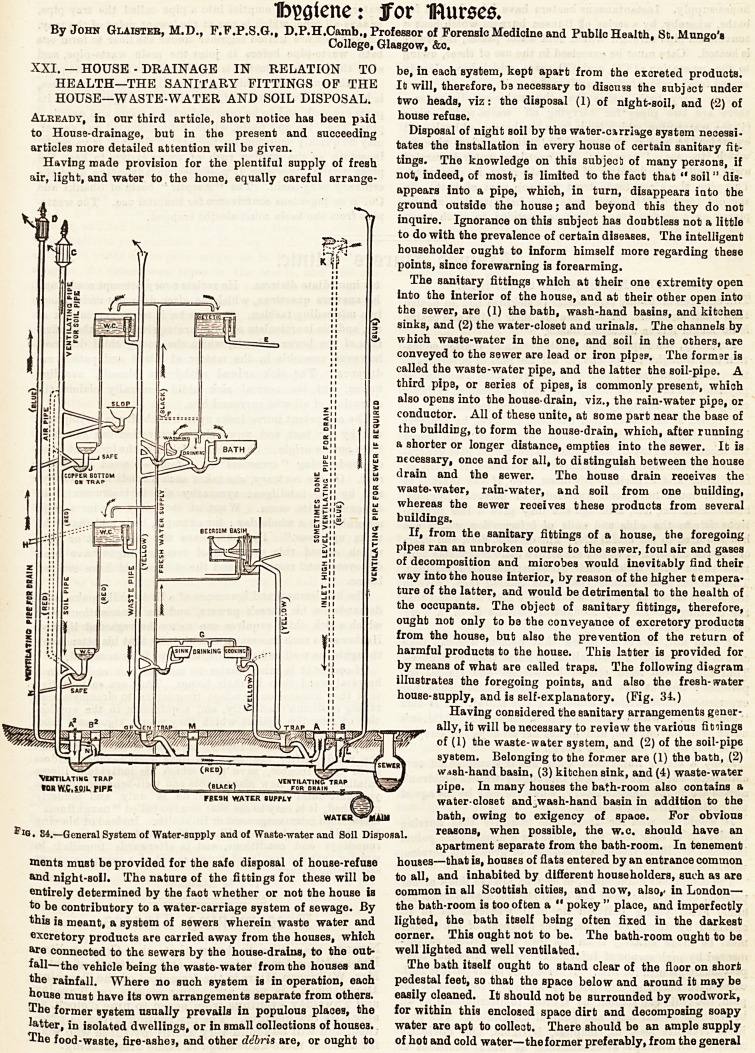# "The Hospital" Nursing Mirror

**Published:** 1896-08-29

**Authors:** 


### "The Hospital" Nursing Mirror.

Being the Extra Nursing Supplement of "The Hospital" Newspaper.

[Contributions for this Supplement should be addressed to the Editor, The Hospital, 428, Strand, London, W.C., and should have the word "Nursing" plainly written in left-hand top corner of the envelope.]

### News from the Nursing World.

### THE DUKE AND DUCHESS OF FIFE AT BANFF.

The patients at Chalmers' Hospital, Banff, had the pleasure of quite a long visit from the Duke and Duchess of Fife a week or two since. The Duke and Duchess spent some time inspecting all the wards, and speaking to many of the patients. Before leaving, the Duchess distributed among them a number of illustrated papers and magazines, as well as welcome gifts of fruit and flowers.

### THE SUPERANNUATION BILL.

It is most unfortunate that the Poor Law Officers' Superannuation Bill, which it was not anticipated would become law during the past session, has, in fact, been passed without the addition of certain amendments essential to the interests of nurses. It should be noted that one of the clauses of the Bill runs as follows: "Existing Officers and Servants.—Any officer or servant in the service or employment of guardians or any other authority empowered by this Act to grant superannuation allowances, may at any time within three months after the commencement of this Act signify in writing to such authority his intention not to avail himself of the provisions of this Act, and in that event it shall not be obligatory on him, notwithstanding anything in this Act contained, to make any contributions or submit to any deduction from his salary or wages under this Act, nor shall he be entitled to receive any superannuation allowance, gratuity, or other benefit under this Act." Nurses should, therefore, at once avail themselves of the protection thus afforded them, and write, protesting against their inclusion in the provisions of the Bill as it stands, to the clerk to the guardians of the union in which they are at work. If they do not take this step, they must clearly understand that they will have to submit to deduction from their wages in consideration of the superannuation allowance, the age for retirement being fixed by the Bill at sixty-five, an age at which the majority of nurses have long ceased to act as officers under the poor-law. In The Hospital "Nursing Supplement" for May 2nd, 1896, p. xli., will be found an article dealing with the whole question of the Bill, and its effects upon the interests of nurses.

### AN EIGHT HOURS DAY FOR NURSES.

In a letter signed "A staff nurse in a large general hospital," which appeared in last week's *British Medical Journal*, the following statement was made with regard to the working hours of nurses generally: "There should be three sets of nurses, each working eight hours, as there are at King's College Hospital, London, and in some American hospitals; then we could have lectures on all the branches of our profession with practical experience, and our hospitals would become first-rate training schools." The writer goes on to say that "our days all the year round are twelve to twelve hours and a-half." An increase in staff and consequent shortening of nurses' hours are most desirable and sadly needed reforms, but they will not be hastened by inaccurate statements of present facts. There are not three separste sets of nurses at King's College Hospital, though the hours on duty do not exceed nine on any working day; and though there are too many institutions where a twelve hours' day obtains, such long hours are fortunately not universal. It has been possible for some years past to have "lectures on all the branches of the profession" without waiting for the introduction of the eight hours' system, and the writer will find on inquiry that a strong feeling exists amongst nurses who have worked in those hospitals where the nursing is divided into three "shifts" that it is bad for the patients, and in practice by no means an unmixed advantage for the nurses.

### RUGELEY DISTRICT HOSPITAL.

Miss Philbrick, the matron of the Rugeley District Hospital, is to be congratulated on the success which attended the festivities organised by her in celebration of the twenty-fifth anniversary of the opening of the hospital. The grounds were prettily decorated, and the volunteer band exerted itself to the utmost with great effect during the afternoon. Brisk business was done at the sale of work held by ladies of the neighbourhood in a tent in the garden, and visitors were numerous, the weather being all that could be wished.

### MASTER AND MATRON.

Under this heading we commented recently upon a statement that at the Plymouth Union Infirmary a nurse could not leave the house without the permission of the master and matron, the superintendent nurse not having the power to grant leave. We are informed by Mr. William Adams, clerk to the Guardians, that one of the rules approved by the board for the management of the hospital provides that "the superintendent nurse shall have entire charge of the sick wards and nurses, under the direction of the medical officer as to all medical requirements, and of the master and matron as to all domestic requirements"; that this rule has been read by certain guardians to mean that the superintendent should have power to grant leave is shown by some members of the board holding it to be "inconsistent with the position of the master and matron"; and that, on the matter being recently brought up again by the master, the committee recommended that a copy of the nursing regulations should be sent to the Local Government Board, with a request that they would say whether or no the regulations in question are in accordance with the law. As the rule at present stands, therefore, we take it that the superintendent nurse is considered to have control over her staff on such matters as leave; but the whole case, and the frequent discussions over this simple question of discipline, shows conclusively how impossible it is for the management of an institution to be carried on harmoniously when the authority of its various officers is so ill defined. Until the position of the superintendent of nursing as supreme in her own department is established and clearly understood, a state of more or less constant friction can only be expected.

### THE PUBLIC AND THE POOR LAW.

The condition of children under the poor law, with reference to the recent report of the Commission on Barrack Schools, was discussed in the House of Commons last week, and some interesting speeches were made. Especially Mr. Mundella made a strong reply to Mr. Chaplin's hope that matters were not really so bad as were reported, deprecating the delay of an hour in bringing these schools under the inspection of the Education Department. In the course of the debate reference was made to the want of trained nurses in workhouses, Major Pryce-Jones expressing a hope that something further would be done in this direction. In reply, Mr. T. W. Russell said that the Board had done a great deal in recent years to secure the provision of trained nurses in workhouses, and had brought all possible pressure to bear on Boards of Guardians in regard to this matter. It is satisfactory to see that there is now a very much more general awakening of public interest in these important questions, which may lead at last to the long-deferred alterations in the general orders of the Local Government Board, without which no real improvement can be secured.

### A NURSE'S REMARKABLE EXPERIENCES.

An extraordinary tale of mistaken identity comes from South Wales and the Midlands. From the *Western Mail* we gather that last year a nurse was charged at Cardiff with obtaining 5s. by false pretences from a decrepit old woman. The accused persisted in her denial that she was the guilty person in spite of several lady witnesses, who swore positively against her. Ultimately the case was adjourned for the attendance of certain officials from the Madeley Union, in which the prisoner said she had been employed. Among them were Miss Ford, assistant matron of Madeley Workhouse, who, in support of the *alibi*, said the prisoner was engaged as a nurse at that place at the time mentioned. A police-sergeant also stated that he had arrested the prisoner there on a charge of larceny. She was, however, acquitted, but, on acquittal, she was re-arrested and taken to Worcester, where she was again charged with larceny, and acquitted. Again she was arrested and taken to Cardiff on this charge of obtaining money on false pretences, and there again the jury returned a verdict of "not guilty." The Recorder concurred, and expressed the belief that a great wrong had been done the woman, who clearly was not guilty. He also ordered the police to take steps to prevent such prosecutions in the future. The prisoner was, in fact, discharged with flying colours. The crowded court cheered her; the jury subscribed her fare to Birkenhead; the Bar made a collection for her, and offerings in money were lavished upon her. This, however, was not to be her final experience of courts of law. This month she appeared as plaintiff in an action against the master of Madeley Workhouse to recover £70 in respect of money lent. Into the details of this matter we need not enter further than to say that here again the nurse was triumphant, the jury finding for the plaintiff for the full amount claimed. Such reiterated triumphs in the law courts seldom fall to any person's lot, and perhaps the best wish we can offer to this nurse is that she may now at last be free from law and its anxieties. We congratulate her most heartily on her escape from what was truly a persecution, a series of accusations for which her conduct had given no excuse. We congratulate her also, but not quite so heartily, in regard to her recent success. We would, however, urge all nurses to avoid monetary dealings with those in authority over them. The position in which they may place themselves by lending and borrowing is a false one, and not always capable of such explanation as was forthcoming in this case.

### TORQUAY NURSES' INSTITUTE.

A special meeting of subscribers to the Torquay Nurses' Institute was called last week to consider the revision of the existing rules. The principal alteration has been the abolishment of a rule which required that the nurses should belong to the Church of England—a limitation rightly felt on all sides to be unwise, and possibly somewhat of a hindrance to the most effectual working of the charity. It was, therefore, with the unanimous approval of those present at the meeting, including the rector of the parish of St. Mark's, that this rule was done away with, and the revised regulations adopted in their entirety. Dr. Wade is the newly-appointed hon. secretary of the institute. A cheque for £28 7s. 4d. has been received by the institute from Mrs. Rawson, the satisfactory result of a fête lately held in her grounds on its behalf.

### TOTNES WORKHOUSE INFIRMARY.

It is obvious that the affairs at the Totnes Workhouse Infirmary require the institution of a Local Government Board inquiry, and that without loss of an unnecessary day, in the interests alike of the nurses and the patients. Readers will remember that the nurses at Totnes were supplied through the Workhouse Infirmary Nursing Association, and that difficulties arose in consequence of their being required to receive and bathe the tramps, an entirely indefensible practice, to which the nurses rightly objected. The Board have declined to rescind this rule, and the reports in the local papers of the various meetings held to consider the matter are instructive reading, showing as they do the entire inability of the guardians to recognise their obligations towards the paupers under their care or to the nurses in their employ. In the ultimate result the nurses, owing to the treatment they received at the hands of the Board, entirely declined to remain in the workhouse, and the guardians refused to pay the assistant nurses, who had only been there for one month, their salaries, on the ground that the association had undertaken they should remain for three months. This was so, but the association could not, even had it wished to do so, compel the nurses to remain under the circumstances, all three being ill from the effects of the bad food supplied to them, and one nurse having actually had to buy the soda and milk ordered for her by the doctor. The guardians also refused a testimonial to one of the nurses, whose interests will, however, fortunately, not suffer from this injustice, the Local Government Board having sanctioned her appointment to another infirmary.

### Hygiene: For Nurses.

By John Glaister, M.D., F.F.P.S.G., D.P.H.Camb., Professor of Forensic Medicine and Public Health, St. Mungo's College, Glasgow, &c.

### XXI. — HOUSE - DRAINAGE IN RELATION TO HEALTH—THE SANITARY FITTINGS OF THE HOUSE—WASTE-WATER AND SOIL DISPOSAL.

Already, in our third article, short notice has been paid to House-drainage, but in the present and succeeding articles more detailed attention will be given.

Having made provision for the plentiful supply of fresh air, light, and water to the home, equally careful arrange-

Fig. 34.—General System of Water-supply and of Waste-water and Soil Disposal.

ments must be provided for the safe disposal of house-refuse and night-soil. The nature of the fittings for these will be entirely determined by the fact whether or not the house is to be contributory to a water-carriage system of sewage. By this is meant, a system of sewers wherein waste water and excretory products are carried away from the houses, which are connected to the sewers by the house-drains, to the outfall—the vehicle being the waste-water from the houses and the rainfall. Where no such system is in operation, each house must have its own arrangements separate from others. The former system usually prevails in populous places, the latter, in isolated dwellings, or in small collections of houses. The food-waste, fire-ashes, and other *débris* are, or ought to be, in each system, kept apart from the excreted products. It will, therefore, be necessary to discuss the subject under two heads, viz: the disposal (1) of night-soil, and (2) of house refuse.

Disposal of night soil by the water-carriage system necessitates the installation in every house of certain sanitary fittings. The knowledge on this subject of many persons, if not, indeed, of most, is limited to the fact that "soil" disappears into a pipe, which, in turn, disappears into the ground outside the house; and beyond this they do not inquire. Ignorance on this subject has doubtless not a little to do with the prevalence of certain diseases. The intelligent householder ought to inform himself more regarding these points, since forewarning is forearming.

The sanitary fittings which at their one extremity open into the interior of the house, and at their other open into the sewer, are (1) the bath, wash-hand basins, and kitchen sinks, and (2) the water-closet and urinals. The channels by which waste-water in the one, and soil in the others, are conveyed to the sewer are lead or iron pipes. The former is called the waste-water pipe, and the latter the soil-pipe. A third pipe, or series of pipes, is commonly present, which also opens into the house-drain, viz., the rain-water pipe, or conductor. All of these unite, at some part near the base of the building, to form the house-drain, which, after running a shorter or longer distance, empties into the sewer. It is necessary, once and for all, to distinguish between the house drain and the sewer. The house drain receives the waste-water, rain-water, and soil from one building, whereas the sewer receives these products from several buildings.

If, from the sanitary fittings of a house, the foregoing pipes ran an unbroken course to the sewer, foul air and gases of decomposition and microbes would inevitably find their way into the house interior, by reason of the higher temperature of the latter, and would be detrimental to the health of the occupants. The object of sanitary fittings, therefore, ought not only to be the conveyance of excretory products from the house, but also the prevention of the return of harmful products to the house. This latter is provided for by means of what are called traps. The following diagram illustrates the foregoing points, and also the fresh-water house-supply, and is self-explanatory. (Fig. 34.)

Having considered the sanitary arrangements generally, it will be necessary to review the various fittings of (1) the waste-water system, and (2) of the soil-pipe system. Belonging to the former are (1) the bath, (2) wash-hand basin, (3) kitchen sink, and (4) waste-water pipe. In many houses the bath-room also contains a water-closet and wash-hand basin in addition to the bath, owing to exigency of space. For obvious reasons, when possible, the w.c. should have an apartment separate from the bath-room. In tenement houses—that is, houses of flats entered by an entrance common to all, and inhabited by different householders, such as are common in all Scottish cities, and now, also, in London—the bath-room is too often a "pokey" place, and imperfectly lighted, the bath itself being often fixed in the darkest corner. This ought not to be. The bath-room ought to be well lighted and well ventilated.

The bath itself ought to stand clear of the floor on short pedestal feet, so that the space below and around it may be easily cleaned. It should not be surrounded by woodwork, for within this enclosed space dirt and decomposing soapy water are apt to collect. There should be an ample supply of hot and cold water—the former preferably, from the general house supply. Instantaneous heaters have been devised for baths, whereby, by a series of Bunsen burners which heat a convoluted pipe through which cold water passes, the water is heated. Care must be exercised in the use of these, owing to the generation of $CO_2$ and CO gases, which have already caused fatal accidents from suffocation.

From every bath—other than the water-supply pipes—there are two pipes for carrying off waste-water, viz.: (1) the waste-pipe, which leads from the bottom of bath, and by which the bath is emptied; and (2) the overflow pipe, by which, in case of overflow, the overflow water is carried away, and thus flooding of the bath-room is prevented. This pipe, therefore, leads from the top part of bath. In the old form of "built-in" bath, the bath itself is usually placed on a leaden tray, which is intended to catch stray water. This tray empties into a pipe called the tray-pipe, or save-all pipe, which is put at the lowest point of the tray. These pipes are united together under the floor to form one bath waste-pipe before it joins the main waste-pipe, and after the junction, must be trapped from the main waste-pipe.

Wash-hand basin: This fitting, to all intents and purposes, is but a miniature bath, and, like the bath, ought to have its parts exposed, and to be provided with a waste and overflow pipe. The "tip-up" basin, which, being mounted on a swivel, may be quickly emptied by the act of tipping, is valuable, inasmuch as the sudden rush of water assists to keep the pipes clean; otherwise, it possesses no advantage over the ordinary plug-basin. The "Aseptic" basin of Shanks and Co. is an ingenious contrivance for hospital use. The waste-pipe from the basin must also be trapped.

### Trained Nurses' Clinic.

### XI.—THE NURSING OF CHILDREN.

The children of to-day fare better in sickness and in health than their ancestors, for the care of their bodies and brains is no longer left in uncertain hands.

Formerly, the impecunious gentlewoman of limited education was thought competent to fill the post of governess to the rising generation, but now the requisite standard of physical and mental efficiency can only be attained satisfactorily by the thoroughly-trained certificated teacher.

Progress in the school-room has been followed by progress in the nursery, and that important apartment is no longer the inconvenient top or back room with which past generations were content. The children's nursemaid is often an intelligent and experienced woman, who, if she intends to succeed, must have some knowledge of personal hygiene, house sanitation, &c., although she may give a less pretentious title to the odds and ends of information which she has acquired in her useful life. The clothes, diet, and exercise of the children of to-day are all of a more reasonable type than those with which their grandparents were familiar. There seems every chance that "the coming race," save in the houses of the very poor, will enjoy a good time, and that the training provided by thoughtful parents is of a very excellent description.

It does not, however, follow that persons who can take care of sound children are qualified to nurse them in sickness. That may often need special knowledge and special experience; but, at the same time, neither knowledge nor experience suffice without something else added. It is this "something" which distinguishes a successful children's nurse from anyone else, and makes her an object of envy to those who do not possess her secret.

Many a clever nurse fails when she is transplanted to a children's ward, but very few thoroughly-trained children's nurses are found unsatisfactory, whatever may be the age of the patient entrusted to them.

It would be well for those who consider that nursing children is an easy task, to consider for a moment the points in which it differs from the treatment of grown-up persons, whether at home or in hospital.

The competent nurse must, of course, always think of her patient, but in the case of the child she has also to think for him. From young children no help whatever is to be obtained by doctor or nurse. The diagnosis of the former must be made entirely from his own observation, supplemented by such scraps of information as the nurse may have previously gleaned for him. Whereas the adult patient indicates with decision, and possibly accuracy, the seat of his pain, the child can seldom be trusted to discriminate at all between an actual ache and general discomfort. The poor little creature feels ill, helpless, irritable, and obviously considers all attempts at examination as wilful additions to his immediate distress. He resists every attempt made, and he answers questions, which he often does not understand, in a misleading fashion. Perhaps he is too young to talk at all, and his inarticulate sorrows are equivalent to the sufferings of the lower animals, whom the young child does not, however, resemble in the matter of silent and patient endurance. The sick animal withdraws himself, avoiding notice, but the normal sick child generally claims the attention of all who surround him.

The competent nurse looks upon irritability as a symptom, and by her tact and unwearied observation can generally trace out its origin. She does not fall into the ignorant error of condemning "crossness" as a fault—a weed without a root. On the contrary, she takes such a condition seriously, and by her intelligent sympathy she is instrumental in discovering its cause. Want of tact may render a child miserable for a whole day; for example, he is told that "he woke up cross." By timely care the discomfort or pain which caused this symptom of temper might have been discovered and remedied, and the child would have escaped blame.

The helplessness and ignorance of a little child make large demands on his nurse's powers, and the conscientious care which a sick child requires can never be regarded lightly. He demands such incessant watchfulness that his attendant's thoughts, as well as her hands, are kept fully occupied.

Of course it is much easier to manage sick children in a hospital ward than in their homes. They are so imitative that they unconsciously adapt themselves to circumstances, taking medicine obediently, and acquiescing in the application of remedies against which they would vigorously rebel if their parents suggested them. Wilful and neglected outside, the child becomes rapidly amenable to discipline within the ward. After the first few days a new-comer seldom gives any trouble, save that which his individual illness entails. Seeing how manageable and well-behaved even the youngest child becomes when he is duly and tenderly cherished, it is easy to see that most so-called "naughtiness" arises from mismanagement or ill-health. Instead of blaming the child a good nurse will consider fairly its general surroundings and conditions, and is afterwards impelled to wonder that the results of both are not of a more disastrous and lasting nature.

*(To be continued.)*

### Dressmaking Classes.

Nurses may like to know that the Cosmopolitan Dresscutting Association, 65a, Oxford Street, of which Mrs. Wilkinson Miers is the Principal of the Teaching Department, is now arranging classes on Tuesday and Thursday evenings from seven to nine o'clock, at specially low charges. The instruction given is practical, and will be found very useful by those who wish for a little more knowledge in home dressmaking.

### Indian Nursing Service.

FROM A CORRESPONDENT.

THE trooping season is drawing near, and in September, perhaps, one or two recruits from home may be going out to join the service. To these I shall give a few hints that may be useful as to what to take out in the way of clothes, &c. Uniforms and patterns of uniforms may be got at Harvey Nicholl's, Knightsbridge. In the way of collars, no turned-down ones are allowed, straight linen collars such as "The Guard's" are worn under the red bands, the uniform white linen cuffs are "The Latest Squire"; these have to be worn, but are not included in the contract. White dresses can be got in India at a reasonable price, but the grey serge, buttons, and red facings are not to be got there. In addition to the uniform you purchase at Harvey Nicholl's, take out at least 1¼ yards of the grey serge, as in India in the cold weather the sisters wear capes in the wards, and you can get them made very cheaply there; also at least quarter-yard of uniform red cashmere—half yard would not be too much—as the collars and cuffs, of red, soon wear out, and you can replace them yourself. The caps are quite easily made, and I should advise you to make about six additional ones to take out. Also half dozen pairs of white bonnet strings; they are 3½ inches wide, and have one tuck and a hem. Flannelette nightgowns are most useful for the winter time, thin cotton for the summer; flannel gets very soon moth-eaten. Don't take out more flannel things than you can possibly help. I never wore a flannel petticoat during my five years in India, but some people do require them, so two would be quite enough. Two nice cotton dressing-gowns, I myself always use Tussore silk; they are cool and wash well. Silk petticoats are very useful, but you can get them cheaper in India, Washing is a mere trifle in India and no consideration, so you had better take at least six white petticoats; you can wear no others in the hot weather; just now at the end of the season they are very cheap. Also the vests now selling at such small prices are worth having. I am taking out one dozen cheap cotton ones and half a dozen of those pretty silk and wool ones, in addition to my old stock. Vests are dear in India. Extra aprons can be bought and made cheaper in India than here, but you must take out plenty to go on with.

Uniform is always worn in India, except for evening dress, when it is optional, and on leave. The most useful dresses you can take out are first and foremost the inevitable black silk, to which have a high and low bodice. A smart coloured silk blouse would be also useful. You can get native silk blouses very cheap in India, and in the hot weather in the plains most people wear simple white mull dresses with low bodices of the same for dinner. I should also recommend a nice tailor-made coat and skirt for your leave, and a few nice shirts. If you ride take out, of course, a riding-habit, but do not have a very heavy one. Most ladies wear washing riding breeches, which you can have made cheaply out there, of Khaki drill, or navy-blue drill. White sailors' hats are worn with uniform, no distinct pattern, but with a plain white ribbon round, and bow at side; better shapes would be got here. On board ship it will be cold as far as Malta; a warm dressing-gown and slippers is indispensable, going backwards and forwards to the bath, and also if you sleep on deck. I use a warm cloak as well, but that is not necessary. Ladies wear evening-dress at dinner on board ship, so take your old ones; black, again, is most serviceable. Your white uniform skirts would do after Suez, with smart blouses.

About your boxes. Write to Curtiss and Sons, Portsmouth or Southampton, for information. They have always acted as my agents, and I find them very economical and most obliging. They have always been very kind to the nursing sisters who have employed them—at least, those I have ever spoken to about it. A lady travelling alone really requires an agent; they give you all information. You can send your boxes to them beforehand, they will meet you at the station, put all your things on board for you, label them and see them in the right parts of the ship, and they will communicate with their agents in Bombay, who will do everything for you there. Their bill they gave me in 1890 was 13s. 8d.; that included carriage of heavy baggage-box from Lowestoft to Portsmouth, meeting me at the station, porterage of my boxes and depositing of them on board, and it saved me pounds of worry and trouble. Do not take out a travelling bath; they occupy a lot of space, and hold very little. Government provides a bath for you, and they are also cheap and plentiful in India.

Wooden boxes are most useful for the hold, any others get smashed up; tin-lined ones are heavy, but considered best. I left my tin-lining in India, on account of the weight, and my things arrived after two months' travelling quite fresh and nice, only an old piece of mackintosh at the bottom of the box and brown paper at the top. Other people have found brown paper alone sufficient. The things I had in a tin box in the hold were all mildewed, the box was damaged and air got in somehow.

Government provides you in your quarters with all necessary furniture, except the following: Pillows, blankets, and bed-linen, table-linen, spoons, forks, knives, table crockery, kitchen utensils, glass, curtains, table-cloths, &c. Take out your own table-napkins, towels, spoons, and forks and knives, a table-cloth or two if you like, and what you will want for your own private rooms; but the cooking utensils, table-crockery, &c., the others will have already bought, and you must be prepared, if asked, to pay a little to the general fund. If you can possibly manage to get ready without taking an advance of money from the Government do so, as it will cripple you for some months in paying it back. You will need at least £5 on board ship with you for accidental expenses. The stewardess expects at least £1, the cabin steward and table steward each 10s. When I came home, the table steward and the rest expected each £1; but we had a long voyage.

The day you arrive in Bombay, if in office hours, go at once to the district staff officer and report your arrival and ask for your "landing certificate." If you have paid your mess bill on board ship, take the receipt for the money (which mind you have got) and go to the pay office. They will pay you from the date of your embarking, which will probably be the day before you sailed; and they will also, if you wish it, give you a month's pay in advance. Take it. If you have not paid your mess bill, they will deduct the messing from your pay. After that, I should go back to the ship and fee the people, &c. You need not disembark and take off your things till the day after arriving, and it will only cost you 2s. for the extra day, and if you were at an hotel 6s. or 7s.; but you would have to arrange the extra day on board with the purser and captain. I always stop at Watson's Hotel, but many people prefer the Apollo Bunder. The higher up your bed-room is at either, the less there is to pay for it. The 6s. ones at Watson's are very good; the floor above is 5s., but a little too high.

About your pillows, if you have not got any of your own, only buy one feather one; get a cotton one in India. For that feather one, in addition to your white pillow-cases, make a nice sateen cover that will not soil easily, and use it for a cushion on board ship and in the railway train, and it will not count as luggage. In Bombay buy a Resai, if you have not an eider-down; it is very like the latter, only stuffed with cotton. It forms mattress, covering, &c.; it should be about 4s. 8d. (English money). You may have some nights in the train going up country, and that, with your pillow, will make you most comfortable.

Get a little basket before travelling up country, or a big basket if you like—no extra charge for tiffin-baskets—with a box of biscuits in—you can buy those on the ship if you like—a flask or bottle with a little lime-juice in, and some fruit will be of great service, also a glass or cup to drink out of. Do not drink plain water travelling, on the trains they sell soda-water very cheap, and you can mix your lime-juice with that. I always carry an enamelled tin tumbler that holds half-a-pint, and then one has the luxury of one's own; they also sell ice on the trains. Some people take a kettle and spirit lamp and make their own tea or coffee, but I always think it too much of a bother, but I have boiled water and filled a hot bottle for my feet when the nights were cold travelling. An indiarubber hot bottle is a great comfort on board ship. Some ladies buy in Bombay a sarahi (or phonetically, sa-rye); you can get one for about four annas or even an anna and a-half; it is an earthenware bottle. If you have your spirit kettle, boil some water and fill it and leave it to cool, it will be quite safe to drink that; water that has been boiled recently is all right. A warm plaid or shawl is a very useful purchase, as everyone travels with their own bedding in India. It looks tidier than blankets, and with a plaid and a resai, unless you went to a very cold winter station, such as Quetta, Peshawar, or Rawal Pindi, you would not need more than one blanket extra, which you would get in India.

### Holidays and Health.

### THE ISLE OF WIGHT.

Many people say that the Isle of Wight is expensive and enervating, and that its appearance is too much like a garden or an inland county in miniature, to be a pleasant seaside place, and they accordingly choose a desolate-looking east coast village to spend their holiday in, leaving the lovely little island to be enjoyed by foreigners, who are more alive to its beauties and its merits. Russians, Greeks, and Germans, especially the last, are to be found there in plenty, and many of the pretty inland places are owned by Greeks, who, like the late Lord Tennyson, enjoy its mild winters and its freedom from snow.

The island presents many different aspects; the bleak Downs of its southern coast protect the land behind them from the rough weather, so that the meadows and trees by their size and luxuriance recall Worcestershire or Herefordshire, and yet within a mile or two the sea is boiling and surging up in huge waves.

A start can be made from Waterloo to Cowes, by way of Southampton, a journey of less than four hours. A few hours at Cowes will suffice, for the place is not particularly interesting, except to those who are in some way connected with the yachts. It is easy to get on by an afternoon train to Freshwater. This is an ideal little place to stay at. Lodgings are somewhat scarce and dear, but the Albion Hotel is comfortable and inexpensive, and there is also a somewhat primitive and rustic Temperance hotel, that is thoroughly comfortable. The beach is not extensive, but the bathing and boating are both good, catching lobsters being an amusement easily combined with the latter. The high chalk cliffs that shut in the little bay are very fine, and contain some curious natural arches and caverns. There are beautiful walks in all directions. The Needles is only three miles off, over the big rolling Downs, and going there by way of Alum Bay, with its many-coloured sand, we get a good view of the Needle rocks and the lighthouse, and of the huge chalk cliffs which rise here almost perpendicularly out of the sea to a height of 600 feet. The peculiar colouring of these intensely white cliffs is seen in no other part of England. They present so flat a surface that there is very little shadow upon them, what there is being of that soft blue hue so rarely seen except upon snow mountains. The effect of these mighty snow-like walls, encompassed by the intensely green sea, and by the clear blue sky, is magnificent, and so vivid as to appear almost "un-English."

Farringford, Lord Tennyson's beautiful home, to which he invited F. D. Maurice in his well-known poem, "though twenty-thousand College Councils" were "thundering anathemas" at him, is close to Freshwater, and its rustic bridge and fine-grown firs are favourite subjects for sketchers and photographers.

Carisbrooke is only twenty minutes by train from Freshwater, and the ruins of the Castle, with its many memories of Charles I., are full of interest; we see the window by which he tried to escape, and the identical iron bar which he filed through with so much trouble, only to find it was too late and that Cromwell's soldiers were waiting for him below. The room where the unfortunate Princess Elizabeth died remains as it was. She was sent to Carisbrooke, and by Cromwell's orders was to be taught the trade of button-making. Not long ago the Queen erected a graceful monument to her memory in Newport Church.

The historic donkey still draws the water for the Castle, and it is amusing to watch the clever manner in which he does his work, walking on and on inside the great wheel, but stopping directly he sees the bucket appear above the well-head, when he trots out of the wheel and thrusts his nose amongst the spectators for pats or biscuits. For the last 600 years the water has been obtained from the deep well in this manner, each donkey having to teach his successor, an instruction which requires about six months.

When the beauties of the immediate neighbourhood of Freshwater are exhausted, a move may be made to Ventnor, a short journey, and there we find plenty of accommodation—indeed, the whole place appears to consist of lodgings. Roseneath, Zig-zag Road, is a comfortable and cheap boarding-house, and Undercliff House, Esplanade, is another equally comfortable one.

Like Freshwater, Ventnor is a capital centre from which to make expeditions. To Shanklin and Sandown, by way of Bonchurch, is a pleasant walk or coach journey; both are pretty little places, though the Chine at Shanklin is much over-rated. The red cliffs between Shanklin and Sandown, as seen from the sands, are interesting. Within a walk, too, is the Roman Villa at Brading. Its mosaic pavements are marvellously perfect, and in the bath-house are still visible the remains of the heating apparatus which helped to make a Roman's bath such a luxury and a pleasure.

Brading Church contains many fine old monuments, and outside it are still to be seen the village stocks and whipping post. Coaches run to most places on the island, so that after the grand and breezy Downs have been explored, there are interesting drives inland to be taken, past many quaint old churches, thatched cottages, and through shady lanes.

The air both at Freshwater and Ventnor is fresh and pleasant, coming, as it does, straight up Channel. A steamer goes from Ventnor to Bournemouth twice a week, and another all round the island for three shillings. This is an excellent way of seeing the cliffs; the different appearance the island presents on its Channel side from that washed by the Solent is interesting to note.

The Consumptive Hospital at Ventnor is a large and well-arranged building, consisting of several distinct blocks, with a chapel in the centre. Visitors are allowed to go over it, and to attend the Sunday afternoon service if they care to do so.

The fare from Waterloo to Ventnor by way of Ryde is 9s. 4d., and the journey takes about four hours.

### Everybody's Opinion.

[Correspondence on all subjects is invited, but we cannot in any way be responsible for the opinions expressed by our correspondents. No communications can be entertained if the name and address of the correspondent is not given, or unless one side of the paper only be written on.]

### THE GRADUATED CHARGES SYSTEM.

"A Michigan Nurse" writes: "The question of payment as above, raised by your correspondent in your issue for June 20th, is really one of great importance to our profession, and I am entirely of her opinion in the matter. If a young woman wishes to be a nurse in the truest and fullest sense, she will consider the pecuniary result a secondary one, and therefore be content to charge, in certain cases, according to her patient's means, though, for the sake of her professional sisters, the patient should be impressed with the fact that the immediate reduction of fee establishes no precedent. Where, therefore, a nurse sees that her patient is practically unable to pay the full fee, I think she is quite justified in not exacting it, and if only she makes it clear that such reduction is a temporary 'act of grace,' I submit that she is as loyal to her profession as she is considerate to her best interests. To those who object to graduated charges, I would suggest the very pertinent fact that, by this system you may often be of material help to a busy doctor, who else might fear to demand the services of a nurse; and my experience is that help so rendered is not forgotten by the doctor when a better case comes up. This, sir, has been my experience since leaving hospital, and I have found no cause to regret such action."

### PROMOTION IN PROVINCIAL HOSPITALS.

A "Provincial Nurse" writes: Will you allow me a space in your paper for a few words on a provincial nurse's grievance? During the last six years only two of the nurses in one of the largest provincial hospitals have been promoted to the post of sister, while sixteen outsiders have had the vacant appointments, two of whom came as staff nurses and had wards given them in a short time; while those who have had three years' hard work for the hospital are overlooked, and year after year seek wards in other hospitals or go in for private work. We know all cannot get wards, and it is said that nurses do not make good sisters in their own training school (if so, we doubt if they would anywhere else!); some have not the capacity of managing a ward or of training those put under them; others, again, may be disloyal to the authorities; but surely out of over seventy nurses some are capable and loyal. If not, how mismanaged the wards must be daily while the sister is off duty, or from Saturday until Monday once a month while she is away for a long Sunday. One would think it a convenience to the medical men to work with nurses whom they know; also a saving in the expenditure, as many nurses would remain on the staff if there was a hope of some day getting charge of a ward in time, and the hospital authorities would not require to get nurses from private institutions so often. It is very discouraging when, after working long and taking much interest in the hospital and its wards, no encouragement is met with from the authorities of our own training school.

### THE TRUTH OF THE MATTER.

"Sister Margaret" writes: Concerning the letter headed "The Truth of the Matter," which appeared recently in your columns, I would like to endorse the sentiments of the writer. There are nurses who have been hindered from taking a three years' course of training, but who nevertheless have worked and studied earnestly, with the result that their knowledge and ability, at any rate, equal their more orthodox sisters. Must these educated and intelligent women be shut out from posts of responsibility for which they may be specially suited, because they have not a three years' certificate? In my own case, not being the happy possessor of home and parents, I was compelled to become a "paying probationer," simply because no vacancy as a regular probationer seemed to exist anywhere without a long period of waiting. It appears to me that each case should be judged on its own merits, for, after all, one nurse may observe and learn as much in one year as another will in three years, and the minds of committees, and even matrons, need to be open to conviction upon this point. The most clever and capable matron under whom I have worked told me that she had only one year's certificate, a fact she regretted, but which certainly did not detract aught from her ability. The very fact that a nurse *is* able to pay for her training usually argues a superior education, a factor of no small account in a profession which demands the highest mental capacity obtainable, though I am very far from thinking that a regular probationer is necessarily less well-educated than a "paying probationer." I only ask, let us all have an equal chance in the keen competition for the highest posts the nursing profession has to offer, and let not a hard and fast line be drawn with regard to a three years' certificate.

### Death in Our Ranks.

With deep regret we hear from Poona of the death of Miss Maud Edger, Nursing Sister, Wanowrse Station Hospital, from cholera, contracted while nursing that deadly disease in the hospital. Miss Edger, who had been working at this station since her arrival in India in 1893, was only twenty-six years of age, and her early death is sincerely mourned by her fellow-nurses, and by many in Poona to whom she had ministered with kindly sympathy in times of illness or injury. The military funeral was very impressive, the coffin covered with the Union Jack and many wreaths and crosses, being carried by non-commissioned officers of the Royal Irish Rifles to its last resting place. General Duncan, General Gatacre, and the colonels commanding the various regiments in the station were present, the chief mourners being Miss Still, the superintendent, A.N.S., and Miss Muir, nursing sister. Two companies of the 2nd Battalion Durham Light Infantry and the bands of the Durham Light Infantry and the Royal Irish Rifles were present.

### Minor Appointments.

West Hampnett Union Infirmary.—Nurse Leeney has been appointed Head Nurse at this infirmary. She received her training at the Chichester Infirmary.

Broomsgrove Union Infirmary.—Nurse Annie Pritchard has been appointed Head Nurse at this infirmary. She was trained at the Wolverhampton Union Infirmary.

Royal Infirmary, Aberdeen.—Miss Elizabeth Davies has been appointed Sister of the medical wards at this hospital. She received three years' training at the Swansea Hospital, and for two years has held the post of sister at the Rotherham Hospital. Miss Davis takes with her many good wishes for success in her new work.

Market Harboro'.—Miss Kate Girdler has been appointed District Nurse at Market Harboro'. She was trained at the General Infirmary, Northampton, and afterwards held the post of head nurse in the women's surgical ward there for two years. Miss Girdler was then appointed charge nurse of one of the large wards at Mill Road Infirmary, Liverpool, with the care also of the operating theatre, which post she held for rather more than two years. Subsequently she took up private nursing in connection with the City and County Institution for Nurses at Worcester. We cordially wish Miss Girdler success in her future work.

Swansea General Hospital.—Miss Agnes E. Sherring has been chosen to fill the vacant appointment of Night Superintendent at this hospital. Miss Sherring was trained at the Royal Southern Hospital, Liverpool, afterwards working at the Men's and Children's Hospital, Southport, and at the South-Eastern Fever Hospital, London; then as sister of the children's ward at the Southampton Infirmary, and as sister of male wards, operating theatre, and out-patient department at the Stanley Hospital, Liverpool. We wish her every success in her new work.

### For Reading to the Sick.

### ON THE CONSECRATION OF OUR MINDS.

**Motto.**

Bringing into captivity every thought to the obedience of Christ.—2 *Cor.* x., 8.

**Verses.**

All thoughts of ill;—all evil deeds,
That have their roots in thoughts of ill;—
Whatever hinders or impedes
The action of the nobler will;—
All these must first be trampled down
Beneath our feet, if we would gain
In the bright fields of fair renown
The right of eminent Domain! —*Longfellow.*

Lord, make my heart a place where Angels sing!
For surely thoughts low-breathed by Thee
Are Angels gliding near on noiseless wing;
And where a home they see
Swept clean, and garnish'd with adorning joy,
They enter in and dwell, and teach that heart to swell
With heavenly melody, their own untired employ.
—*Keble.*

All Thoughts that mould the age, begin
Deep down within the primitive soul;
And from the Many slowly upward win
The One who grasps the Whole.
All Thought begins in Feeling—wide
In the great mass its base is hid,
And narrowing up to Thought, stands glorified—
A moveless pyramid!
Nor is he far astray, who deems
That every hope which rises and grows broad
In the World's heart, by ordered impulse streams
From the Great heart of God. —*Lowell.*

Lord, make these faithless hearts of ours
Such lessons learn from birds and flowers;
Make them from self to cease,
Leave all things to a Father's Will,
And taste, before Him lying still,
E'en in affliction, peace.

**Reading.**

There is nothing either good or bad, but Thinking makes it so.—*Shakespeare.*

Accustom yourself to think upon nothing but what you could freely reveal, if the question were put to you.
—*Marcus Aurelius.*

"Whatsoever things are true, whatsoever things are honest, whatsoever things are just, whatsoever things are pure, whatsoever things are lovely, whatsoever things are of good report; if there be any virtue, and if there be any praise, think on these things."—*Phill.* iv. 8.

"Think on these things"—consider these things, and keep the current of your thoughts set towards them; let your minds be busy with them, and let them tell on all your views of life. Such seems to be the force of the word which St. Paul uses here. He is giving a rule, I believe, with regard to a part of our life and a field of self-discipline which deserves far more care than it often gets. He does not seem to be speaking of thought with an immediate regard to action, for his advice as to outward conduct is given in the next verse; nor is he speaking here of meditation as a religious exercise, for the lines of thought which he would point out seem too wide and general for that. Rather he is telling us, I think, how we ought to set and train and discipline our minds how to use their leisure; how they ought to behave, so to speak, when they are not on special duty.—*Dean Francis Paget.*

### Notes and Queries.

The contents of the Editor's Letter-box have now reached such unwieldy proportions that it has become necessary to establish a hard and fast rule regarding Answers to Correspondents. In future, all questions requiring replies will continue to be answered in this column without any fee. If an answer is required by letter, a fee of half-a-crown must be enclosed with the note containing the enquiry. We are always pleased to help our numerous correspondents to the fullest extent, and we can trust them to sympathise in the overwhelming amount of writing which makes the new rules a necessity. Every communication must be accompanied by the writer's name and address, otherwise it will receive no attention.

**Queries.**

(152) *Disinfection.*—Can you tell me the best way to cleanse and disinfect a room infested with bugs? Would formaldehyde vapour be of any use?—*Dragon.*

(153) *Holiday Engagements.*—A nurse trained in one of the best hospitals in India, with eight years' experience in hospital and private work in India, and good references, is anxious to spend a few months in any English hospitals (London preferred) in taking holiday work. Can you tell her to whom she should write, or how she can make sure of an engagement before leaving India?—*Nurse.*

(154) *Sanitary Inspectorship.*—Where can I study in order to qualify as a sanitary inspector?—*Enquirer.*

(155) *Nursing Abroad.*—I have been advised to go abroad on account of my health, which has been suffering from overmuch night work. Would you advise me to go to South Africa, and can you tell me how to set about obtaining an engagement? I am quite strong now after a long holiday, and fit for work. My age is 30.—*Nurse B.*

(156) *Massage.*—Please tell me what is the best certificate of massage to be obtained in London? Also please tell me about the L.O.S. diploma, what happened after its condemnation by the General Medical Council? Can anyone now get the diploma who is not a medical student?—*Trained Nurse.*

(157) *Nursing in Biarritz.*—Can you give me any information about nursing institutions in Biarritz?—*Doubtful.*

(158) *A Case of Paralysis.*—Please tell me if there is any institution in the neighbourhood of Peckham where such a case could be placed at small cost.—*H. R.*

**Answers.**

*Training* (Miss S.)—Will Miss S., from whom a query under the above heading appeared in this column for August 1st, kindly send her address to the editor.

*Nursing at Ilfracombe.*—C. T. L. is referred to the notice at the head of this column. Anonymous queries cannot be replied to.

*Lectures for Nurses in London.*—Nurse Daisy must send full name and address before her query can be answered.

*Hospital Linen.*—See notice heading this column. We cannot reply to anonymous queries.

(152) *Disinfection (Dragon).*—Mrs. Dacre-Craven, in her "Guide to District Nursing," gives the following advice: "The best remedy for getting rid of vermin is cleanliness, light, and air; without these three factors nothing is of much use. Bedstead and bedding must be well brushed every morning. All cracks and joints of the bedstead must be filled with a thick paste of carbolic powder mixed with a little clear carbolic in solution, and laid on freely with a brush (or the common carbolic may be used). . . . . When bugs infest the flooring, make a paste of chloride of lime or of carbolic powder, and fill all crevices with it between the planks of the flooring and the skirting boards. The bedstead must be kept at least a foot from the walls, and the legs of the bedstead placed on saucers filled with water with a little carbolic in it until the walls and floor are thoroughly disinfected. The blankets and bedding must be sprinkled daily with Keating's insect powder. The wall papers must be stripped off, and for a time the wall should be distempered, and the floor should be washed with solution of perchloride of mercury (1 in 1000)."

(153) *Holiday Engagements (Nurse).*—Your best plan would be to advertise, but we do not think you will find it easy to make any such arrangement while still in India. In the large London hospitals the staff is usually sufficient to allow for holidays, without requiring the services of extra nurses, and probably few matrons would care to engage for that purpose, if required, a nurse unused to the routine of English hospital life. You might write to the matrons of some London and provincial hospitals (of which you will find complete lists in Burdett's "Hospitals and Charities"), but personal application on your arrival in England will be the more likely method of finding what you want.

(154) *Sanitary Inspectorship (Enquirer).*—Write to Miss Lamport, 52, St. John's Wood Road, N.W., or to the Dean of Bedford College for Women, Baker Street, W.

(155) *Nursing Abroad (Nurse B.)*—So far as the climate goes, doubtless you would find South Africa very suitable. Miss Mitchell, matron of the Eaton Convalescent Home, Plumstead, Diep River, South Africa, might be able to help you with advice. We always strongly dissuade nurses from going abroad on the *chance* of finding employment. It is a very serious risk, and too often ends in failure.

(156) *Massage.*—(1.) The Society of Trained Masseuses, 12, Buckingham Street, Strand, hold examinations in massage, and give certificates. This is the only society we know of that examines pupils not trained by its own teachers. Nearly all teachers of massage give some certificate to their pupils after a course of instruction. (2.) The London Obstetrical Society holds its examination as usual every three months, and grants certificates to successful candidates. The certificate is now worded differently; it used to run, "is a skilled midwife, and competent to attend natural labour." It now is, "passed our examination in midwifery." As far as the qualification for a midwife is concerned, there is no practical difference. You had better write for their papers, which will give you all information, or if you wish to make inquiries as to instruction, &c., see the Secretary, at the Midwives' Institute, 12, Buckingham Street, Strand. Neither the course of study nor the certificate have or even had anything to do with medical students.

(157) *Nursing in Biarritz (Doubtful).*—Write to Sister Mary, Lady Superintendent of the English Nurses' Home, Chalet Marcelle, Biarritz. We do not know of any other.

(158) *Case of Paralysis (H. R.).*—We do not know of any such institution in the south-east of London. You will find complete lists of homes and institutions for incurable or chronic cases in Burdett's "Hospitals and Charities."

## Figures and Tables

**Fig. 34. f1:**